# Design and Selection of Novel C1s Inhibitors by In Silico and In Vitro Approaches

**DOI:** 10.3390/molecules24203641

**Published:** 2019-10-09

**Authors:** Katalin Szilágyi, István Hajdú, Beáta Flachner, Zsolt Lőrincz, Júlia Balczer, Péter Gál, Péter Závodszky, Chiara Pirli, Balázs Balogh, István M. Mándity, Sándor Cseh, György Dormán

**Affiliations:** 1 Targetex Biosciences, Madách Imre utca 31/2., H-2120 Dunakeszi, Hungary; szilagyi@targetex.com (K.S.); hajdu@targetex.com (I.H.); flachner@targetex.com (B.F.); lorincz@targetex.com (Z.L.); cseh@targetex.com (S.C.); 2 Institute of Enzymology, Research Centre for Natural Sciences, Hungarian Academy of Sciences, Magyar Tudósok Körútja 2., H-1117 Budapest, Hungary; balczer.julia@ttk.mta.hu (J.B.); gal.peter@ttk.mta.hu (P.G.); zavodszky.peter@ttk.mta.hu (P.Z.); 3 Department of Organic Chemistry, Faculty of Pharmacy, Semmelweis University, Hőgyes Endre u. 7, H-1092 Budapest, Hungary; chiara.pirli@gmail.com (C.P.); balogh.balazs@pharma.semmelweis-univ.hu (B.B.); mandity.istvan@ttk.mta.hu (I.M.M.); 4 MTA TTK Lendület Artificial Transporter Research Group, Institute of Materials and Environmental Chemistry, Research Center for Natural Sciences, Hungarian Academy of Sciences, Magyar Tudósok Körútja 2., H-1117 Budapest, Hungary; 5 Faculty of Pharmacy, Institute of Pharmaceutical Chemistry, University of Szeged, Zrínyi u. 9., H-6720 Szeged, Hungary

**Keywords:** complement system, virtual screening, C1s inhibitor, FactorXa, biological screening, pharmacophore modelling

## Abstract

The complement system is associated with various diseases such as inflammation or auto-immune diseases. Complement-targeted drugs could provide novel therapeutic intervention against the above diseases. C1s, a serine protease, plays an important role in the CS and could be an attractive target since it blocks the system at an early stage of the complement cascade. Designing C1 inhibitors is particularly challenging since known inhibitors are restricted to a narrow bioactive chemical space in addition selectivity over other serine proteases is an important requirement. The typical architecture of a small molecule inhibitor of C1s contains an amidine (or guanidine) residue, however, the discovery of non-amidine inhibitors might have high value, particularly if novel chemotypes and/or compounds displaying improved selectivity are identified. We applied various virtual screening approaches to identify C1s focused libraries that lack the amidine/guanidine functionalities, then the in silico generated libraries were evaluated by in vitro biological assays. While 3D structure-based methods were not suitable for virtual screening of C1s inhibitors, and a 2D similarity search did not lead to novel chemotypes, pharmacophore model generation allowed us to identify two novel chemotypes with submicromolar activities. In three screening rounds we tested altogether 89 compounds and identified 20 hit compounds (<10 μM activities; overall hit rate: 22.5%). The highest activity determined was 12 nM (1,2,4-triazole), while for the newly identified chemotypes (1,3-benzoxazin-4-one and thieno[2,3-*d*][1,3]oxazin-4-one) it was 241 nM and 549 nM, respectively.

## 1. Introduction

**Scheme 1:**
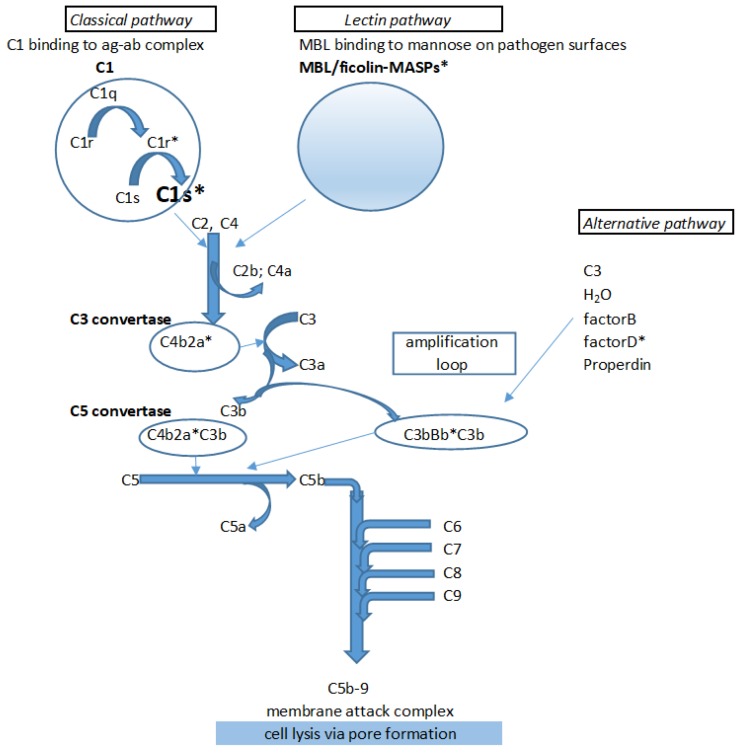
Schematic overview of the activation of the complement system via its activation pathways highlighted C1s, the enzyme transducing activation signal from C1 to the next step in the activation cascade via activating, i.e., cleaving C2 and C4 components.

In our drug discovery project our objective was to identify potential C1s inhibitors, preferably having novel chemotypes as well as with acceptable selectivity over related serine proteases. The complement system (CS) is a key component of innate immunity, which is involved in several physiological and pathological processes. Dysregulated or impaired complement is involved in an increasing list of human diseases (e.g., autoimmune, inflammatory, and neurodegenerative diseases etc.). CS consists of over forty protein components that are present in the blood or on cell surfaces. The CS is activated by infection or by injury. Activation may be prolonged or misdirected to healthy cells and can lead to inflammatory or auto-immune diseases. Complement-targeted drugs could provide novel therapeutic intervention against the above diseases. Nine serine proteases are integral elements of the CS cascade (C1r, C1s, C2, MASP-1, MASP-2, MASP-3, factor D, factor B, factor I–Scheme 1). C1s is present as a proenzyme within the C1 complex, the first component of the CS consisting of a C1q, two C1r and two C1s molecules. The activation of the C1 complex is the first step of the classical activation pathway of CS, which is initiated by the interaction of C1q with immunoglobulin (Ig) antigen complexes. The activation signal is mechanically transmitted by C1q to C1r dimers; activated C1r proteases then cleave and activate the C1s proenzymes. Activated C1s protease forwards the activation signal by cleaving C4 and C4b-associated C2 to form C3 convertase C4b2a, so an inhibitor that targets the C1s protease domain could block the activation of the classical pathway of CS [1,2].

The main challenges in the C1s inhibitor discovery are the following: (1) the very distinct, narrow bioactive chemical space of the available inhibitors, and (2) the selectivity over other serine proteases (including the proteases of the blood coagulation and fibrinolysis etc.). While there are several peptide-based C1 system inhibitors [3] small molecules are relatively rare and non-selective. The typical architecture of a small molecule inhibitor of C1s contains a heterocyclic amidine (or guanidine), such as nafamostat (FUT175) [4], which inhibits several serine proteases, thus, it is uncertain that its physiological effects are associated with the complement system. Janssen researchers reported arylsulfonylthiophene-2-carboxamidine inhibitors [5,6,7] that exhibited somewhat improved selectivity. Due to poor pharmacokinetics, introduction of pegylated linkers led to related compounds with good potency and in vivo pharmacokinetic properties [8]. Recently, various inhibitors lacking the amidine (guanidine) “warheads” were identified by HTS technologies (Molecular Library Screening Center Network (MLSCN); Penn Center for Molecular Discovery (PCMD); listed in PubChem) [9,10] and reported by Chen et al. [11]. Due to the limited availability of the amidine and guanidine derivatives from commercial repositories we have chosen the reported O, N-heterocycles (lacking the “warheads”) and the potential bioisosteric replacements of amidine (guanidine) motifs as starting points for selecting C1s focused compound libraries.

Over the past two decades huge compound repositories were built exploiting historical collections as well as compound libraries. At the same time the chemoinformatics methods have developed rapidly, the computational power increased allowing fast or real time calculations. In addition, deeper knowledge has gathered about the small molecule–protein interactions using state-of-the-art X-ray crystallography, docking and 3D modelling [12]. In our efforts to identify novel C1s inhibitors we intended to use this huge available commercial chemical space and virtual screening (VS) using various approaches (2D similarity search, 3D modelling and pharmacophore model building).

Virtual screening leading to focused libraries has become a popular technique [13] since it was expected to reduce the synthesis and biological screening cost and shortens the life cycles of the discovery phases. Furthermore, focused library screening often results in many fold increases in the hit rate compared with random screening of commercial libraries [14,15].

From its emergence vs. combined 2D/3D ligand-based and structure-based approaches [16,17]. The overall size of the existing compound collection is estimated over 35M based on the Zinc library [18,19]. eMolecules [20] contains 7 million commercially available, unique structures from various library providers. In our library selection process, we used a 5 million collection of commercially available compounds collected from the most relevant suppliers [21,22,23,24,25,26,27,28].

Measuring the C1s inhibitory activity we adapted a photometric and fluorescent assay and validated the assay with known C1s inhibitors. The protein was expressed in a bacterial system.

For considering selectivity of the tested C1s inhibitors, Factor Xa—a key component of the coagulation cascade—was selected. Coagulation or clotting system is an analog serine protease cascade, regarding numerous serine protease elements, various pathways of activation, multiple points of regulation, with a potential amplification loop. Factor Xa is an activated form of Factor X, a key component of the coagulation system, a trypsin-like serine protease holding a central position that links the intrinsic and extrinsic activation pathways. FXa is similar to C1s considering its modular nature; activation by site-specific cleavage; containing a serine protease (SP) domain with strikingly similar binding sub-sites, however, in C1s large insertion yields higher degree of selectivity with concomitant decrease in activity [29,30]. Furthermore, C1s and Factor Xa have similar active site substrate specificity [31,32].

## 2. Results

### 2.1. Generation of C1s Inhibitor Focused Library (by 2D Methods)

**Scheme 2:**
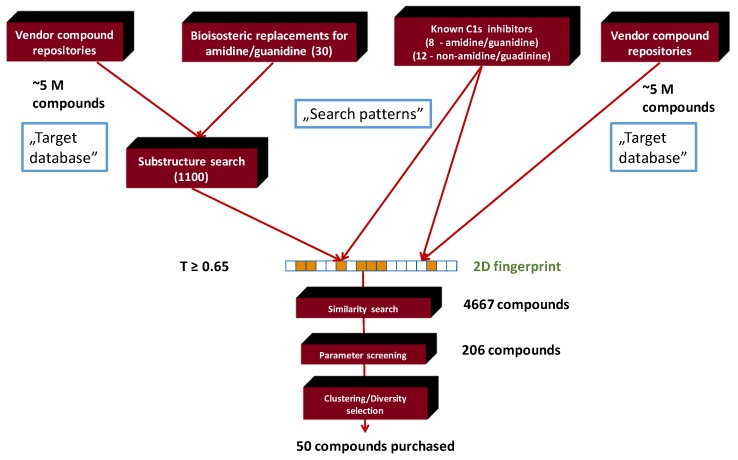
Flow chart of the focused library generation by 2D similarity search.

We first collected known C1s inhibitors from PubChem and available publications, as well as generated the bioisosters of the key recognition motifs (guanidine, amidine). Based on the resulting novel structural motifs and the structure of the known inhibitors (“Search patterns”) a focused library was selected from commercial vendor libraries (5 million compounds, “Target database”) upon 2D structural similarities (Scheme 2).

Known C1s inhibitors that lack the amidine/guanidine moieties were identified in PubChem and selected publications. After clustering the various inhibitors that have low μM inhibitory activities, and removing the redundant structures eight amidine/guanidine and 12 non-amidine/guanidine compounds were selected as seed/reference compounds for 2D similarity search (Supplementary Materials Table S1a,b).

For generating the bioisosteric replacements of the amidine and guanidine recognition motifs we applied literature sources [33] and software [34] (30 selected bioisosteric transformations can be found: Supplementary Materials, Scheme S1). Relatively few compounds (1100) were found in the vendor databases that contained the generated bioisosteric replacements and those compounds were also used for similarity search matching their structures with the reference compounds. The two searches resulted in 4667 compounds applying T ≥ 0.65 Tanimoto coefficient as a similarity cut-off value.

Virtual focused library generation is often linked to target families that represent a distinct chemical, biological and property space [35]. Several target families have a different property range, therefore, defining a target-specific parameter space is often more practical for focused library filtering than applying the standard Lipinski’s Rule of 5-based filtering [36]. Those rules propose general limits (cut-off values and ranges) for library filtering and represent a parameter “window” favorable for oral administration.

Physico-chemical parameter determination of the known C1s inhibitors are the following (amidines, guanidines were excluded): Mol. weight = 213–358 Da; LogP = 1.67–4.15; tPsa = 35 – 80 Å^2^; H-bond acceptor = 2–5; H bond donor = 0–1; rotatable bond = 1–5. Thus, the initial C1s focused library was filtered for the above physico-chemical parameter space of the known inhibitors leading to 206 structures. Finally, 50 compounds were selected for purchase based on cluster representation and diversity.

### 2.2. Generation of C1s Inhibitor Focused Library (Pharmacophore Search Methods)

**Scheme 3:**
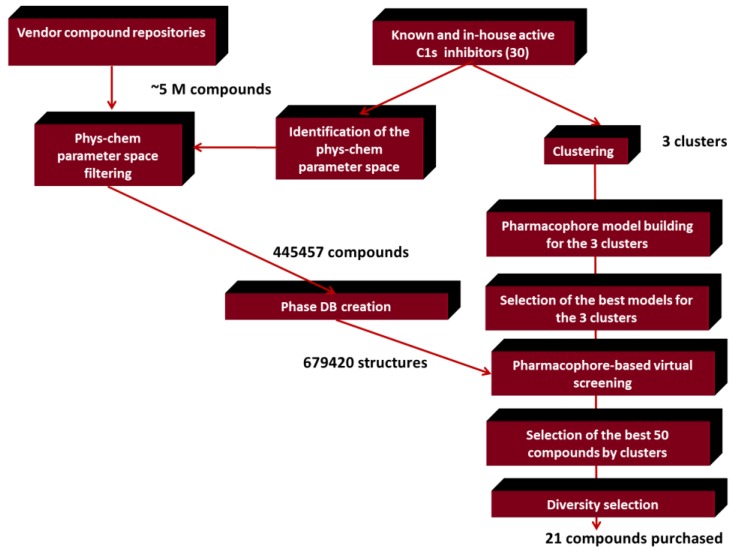
Flow chart of the pharmacophore–based virtual screening and focused library generation.

Physico-chemical parameter space filtering of the 5 million compound vendor libraries applying the previously identified ranges except the molecular weight range was set wider (200–450 Da); removal of the duplicated structures resulted in 445,457 compounds (Enamine (71,311 compounds), ChemDiv (258,797 compounds), Chembridge (60,052 compounds), Interbioscreen (31,255 compounds) and Life Chemicals (24,042 compounds). In the next stage, phase database creator (including ligand preparation up to 100 conformers and stereoisomers ionization and tautomers) was used to develop pharmacophore libraries leading to 679,420 structures that were used as entries in the pharmacophore-based virtual screening (Scheme 3). The virtual screening was carried out by the three major clusters representing the 2D focused library screening hits (1,2,3-benzotriazoles, 1,2,4-triazoles, 3,1-benzoxazin-4-one) applying the best models selected for each cluster.

Phase ligand and database screening task was used to identify new candidates applying the best models with pre-screen using database keys, the maximum number of features and 1.0 Å distance tolerance options. Hits were ranked by Phase Screen Score and only one hit per molecules was kept.

Compounds having the highest 50 phase scores were selected for the three compound clusters each (3 × 50).

Cluster I contains compounds having the highest phase score ranges coming from the 3,1-benzoxazin-4-one-based pharmacophore model (best 50: 2.94–2.55); Cluster II. contains compounds having the highest phase score ranges coming from the 1,2,3-benzotriazole model (best 50: 2.81–2.35); while Cluster III contains the compounds having the highest phase score ranges coming from 1,2,4-triazole model (best 50: 2.54–2.32). Based on the phase scores and maintaining the maximum structural diversity we selected 21 compounds for biological screening (nine compounds from the best model for 3,1-benzoxazin-4-one; seven compounds from the best model for 1,2,3-benzotriazole and five compounds from the best model for: 1,2,4-triazole).

### 2.3. Generation of C1s Inhibitor Hit-Validation Small Library

For hit validation 11 thieno[2,3-*d*][1,3]oxazin-4-ones and seven 1,3-benzoxazin-4-one derivatives were selected as close analogues of the pharmacophore hit compounds from commercial databases. A simple substructure search resulted in ca. 350 compounds having the thieno[2,3-*d*][1,3]oxazin-4-one core and only 50 compounds containing the 1,3-benzoxazin-4-one scaffold. Based on the substitution pattern of the hit compounds the close analogues were selected by visual structural analysis.

### 2.4. In Vitro Screening Results

#### 2.4.1. C1s Inhibition

Table 1, Table 2, Table 3, Table 4 and Table 5. shows the C1s inhibitory activities according to the 5 chemotypes: 1,2,3-benzotriazoles (Table 1); 1,2,4-triazoles (Table 2); 3,1-benzoxazin-4-ones (Table 3); thieno[2,3-*d*][1,3]oxazin-4-one (Table 4); 1,3-benzoxazin-4-one (Table 5).

#### 2.4.2. Factor Xa Inhibition

Out of the 20 C1s hit compounds 6 compounds exhibited significant selectivities towards C1s (Table 6, remaining activities are > 85% in the Factor Xa assay).

## 3. Discussion

In our drug discovery project, the first attempt was to generate a C1s inhibitor-focused library applying 2D similarity searches on multimillion compound commercial libraries based on the structure of known inhibitors collected from PubChem and literature sources. Many C1s inhibitors contain amidine (or guanidine) moieties binding to Asp611 [5]. However, compounds with such functionalities are rarely available from commercial vendor libraries, therefore, we turned to the generation of their bioisosteric replacements and other recently published inhibitors that lack such structural units. The PubChem and literature seed compounds (12) are composed of four chemotypes: 1,2,3-benzotriazoles (4), 1,2,4-triazoles (4), imidazoles (3), and 3,1-benzoxazin-4-ones (1). The performance of the 2D similarity search relies on the diversity of the seeds and the searchable chemical space (vendor libraries), the computational methods (fingerprints) as well as the applied criteria (similarity cut-off values, and property space filters etc.). Various research groups have analyzed large activity data sets and come to the conclusion that on average there is a 30% probability that two compounds shared the same activity within a certain similarity cut-off (Tanimoto coefficient ≥ 0.85) [37,38,39]. However, applying T ≥ 0.65 cut-off value the virtual hits still represent high level of acceptable similarities. On the other hand, such structural similarity does not necessarily lead to novel chemotypes or scaffolds, which is one of the major challenges of the contemporary drug discovery [40].

One of the critical issues in 2D ligand-based similarity selection approaches is the novelty vs. similarity [41], thus, structural analysis of the hits is a necessary task after biological evaluation of the focused library. Novelty could mean different core structure (scaffold/chemotype) or in the case of linear compounds a different arrangement of the various motifs although in a different substitution pattern [42]. After diversity selection and visual inspection, we purchased 50 compounds, which contained 1,2,3-benzotriazoles (16), 1,2,4-triazoles (15), imidazoles (6), 3,1-benzoxazin-4-ones (5) and five unrelated (novel) chemotypes (see Supplementary Materials, Tables S2–S4) at T ≥ 0.65 Tanimoto similarity level. We did not go under this threshold, which might have significantly reduced the hit rate. Indeed, biological screening led to a relatively high hit rate: 22%, however, did not reveal novel chemotypes. The distribution of the hits was 1,2,3-benzotriazoles (6), 1,2,4-triazoles (4), imidazoles (only one weak hit), 3,1-benzoxazin-4-ones (1).

The hit structures were closely related to the seed compounds but revealed new analogues with different substituent pattern and improved C1s inhibitory activities. The two highest activity compounds (#9 and #10) belonged to the 1,2,4-triazole cluster (IC_50_ = 12 and 44 nM).

**Figure 1:**
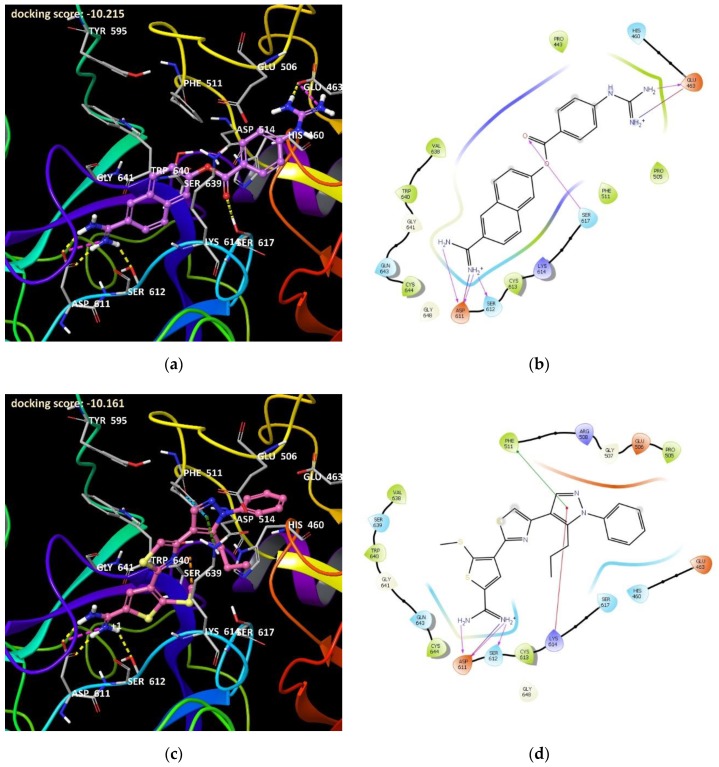
Docking results with the optimized 1ELV structure. Best docking pose (**a**) and ligand interaction diagram (**b**) for compound FUT-175; and best docking pose (**c**) and ligand interaction diagram (**d**) for Subasinghe’s compound 49. Both inhibitors’ amidine group formed a salt bridge with Asp611 and a further H-bond with Ser612. In case of FUT-175 a second salt bridge was formed between the guanidine group and Glu463 and an additional H-bond with the catalytic Ser617 was also observed. Compound 49 was stabilized through its pyrazole ring forming both a π-π stacking interaction with Phe511 and a π-cation interaction with Lys 614.

In order to identify C1s inhibitors with novel chemotypes we turned to building 3D binding and pharmacophore models. 3D modelling/pharmacophore. The docking studies with the unmodified 1ELV structures resulted in relatively low scores for the most active Subasinghe amidine compounds (with K_i_ > 1 μM [5]) and very low scores for the non-amidine type (Chen [11] and 2D focused library hit compounds) and especially low scores for the FUT-175, a high activity C1s inhibitor (IC_50_ = 29 nM) published by Aoyama et al. [4] in 1984, and often used as a reference compound. The saltbridge indicated by Subasinghe between Asp 611 and the protonated amidine groups were observable for most of the poses but the other parts of the ligands were disposed. The scores for the non-amidine compounds (Chen [11] and 2D focused library hits) were even lower. Our hypothesis was that the orientation of the amino acid side chains in the binding site was not optimal for the binding, and that is why we decided to improve the geometry of the site with induced fit docking (IFD) approach using the most active Subasinghe thiopheneamidine inhibitor: compound 49 (K_i_ = 60 nM [5]). The re-docking of compound 49 in [5] and the docking of the FUT-175 reference compound into the IFD improved 1ELV structure resulted in significantly higher scores (−10.215 and −10.161) and better poses (Figure 1a–d). These values corresponded to the biological activity values.

All 39 Subasinghe’s thiopheneamidine inhibitors [5] were successfully docked into the IFD 1ELV structure resulted in a better alignment and significantly higher scores (between −7.5 and −10.5). No strong correlation between the biological data and the docking scores could be observed, but the inactive compounds were given lower scores while only a few active compounds were underscored (see Supplementary Materials). The data indicated that the docking might be able to identify the C1s active compounds in the course of an in silico screening.

As a next step our validation, a number of biologically active but chemically diverse C1s inhibitors were also docked. The first group of compounds was identified as hits of the 2D similarity-based focused while the other group of compounds was published by Chen et al. [11] as a result of a virtual screening study. The Glide docking scores (empirical scores of binding affinity expressed in kcal/mol) for both groups of non-amidine compounds were significantly lower than expected (between −5.0 and −7.0). The second group also contained a number of inactive compounds (IC_50_ > 50 μM), these were predicted scores comparable with those of the active ones (Table 7 and Table 8).

These results indicate that our model might not be able to identify other than amidine inhibitors. The gap between the biological data and the scores could be a result of the lack of our knowledge on the C1s small molecule binding. We assumed these compounds were blocking the access of the protein substrates toward the catalytic residues and we were focusing on the catalytic Ser617 as the obvious location of the inhibitor binding. This seems to be reasonable in the case of the amidine compounds considering that the distance between Asp611 and Ser617 is comparable with the size of these inhibitors. However, while the dockings are indicating that the salt bridge with Asp611 could be a key factor in the case of the Subasinghe compounds’ and FUT-175 binding, it was hard to find something equally strong in case of the non-amidine compounds and it is quite possible that the binding mode of these inhibitors are fundamentally different. It is also possible that they bind in another location further away from Ser617, which we have not investigated, and they are blocking the enzyme with a different mechanism. Thus, we do not know the exact location of the potential inhibitor binding site and the mechanism of the inhibition and we have no coordinates of inactive enzyme to compare with our results. Unfortunately, no docking study other than for the Subasinghe amidine inhibitors [5] has been reported so far for the C1s enzyme. In conclusion, 3D model validation resulted in negative results providing low docking scores and the models did not discriminate the active and non-active structures for non-amidine inhibitors.

These uncertainties prompted us to use a different approach instead of docking in the virtual screening, and this is why we turned to a pharmacophore-based method. Recent studies on pharmacophore based drug discovery in silico studies were reported where ligand- and structure based approaches were successfully applied [43,44,45]. The number of published human C1s inhibitory data in the literature is very limited as well as the suitable target structures, therefore we were focusing a purely ligand based method. The largest group was the above mentioned amidine compounds investigated by Subasinghe et al. [5,6,7]. For practical reasons we were focusing on different kind of compounds.

After a comprehensive database survey three sources with large number of non-amidine active compounds were identified. The first source of data was a journal (*Biomolecules*) article published by Chen et al. [11]. The authors tested 17 compounds and nine of them were found to be active, including 1,2,3-benzotriazoles (5) and phenylfuran esters (4). The second group of active compounds were found in an unpublished bioassay AID 538 and 787 [9,10] from the PubChem database [46]. A total number of 184 compounds were tested, 23 of them proved to be active including 1,2,4-triazoles (6), 1,2,3-benzotriazoles (5), imidazoles (4), 3,1-benzoxazin-4-ones (2) and other structures (6). The third set of compounds was the collection based on our 2D in silico selection from various commercial sources (see above). Twelve out of the 50 tested compounds were active, including 1,2,3-benzotriazoles (6), 1,2,4-triazoles (4), 3,1-benzoxazin-4-ones (2) and an imidazole.

**Scheme 4:**
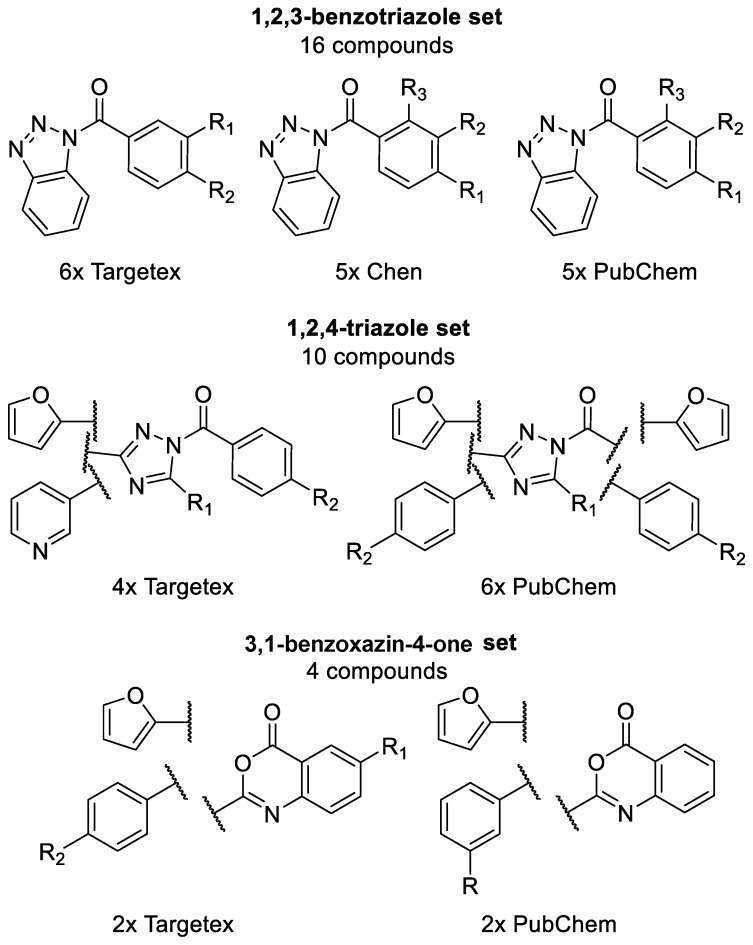
Known C1s inhibitors used as input compounds for pharmacophore model building (“Targetex” means hit compounds of the 2D in silico selection: see Table 1, Table 2 and Table 3.).

Based on common scaffolds, these data were merged into three sets of structurally related compounds, a 1,2,3-benzotriazole set with 16 active compounds, a 1,2,4-triazole set with 10 active compounds and a 3,1-benzoxazin-4-one set with four active compounds (Scheme 4). The imidazole compounds were structurally diverse so it was impossible to build a fourth set for consensus pharmacophore modelling. The inactive compounds were used as a ‘decoy’ set in the pharmacophore validation during the process.

**Figure 2:**
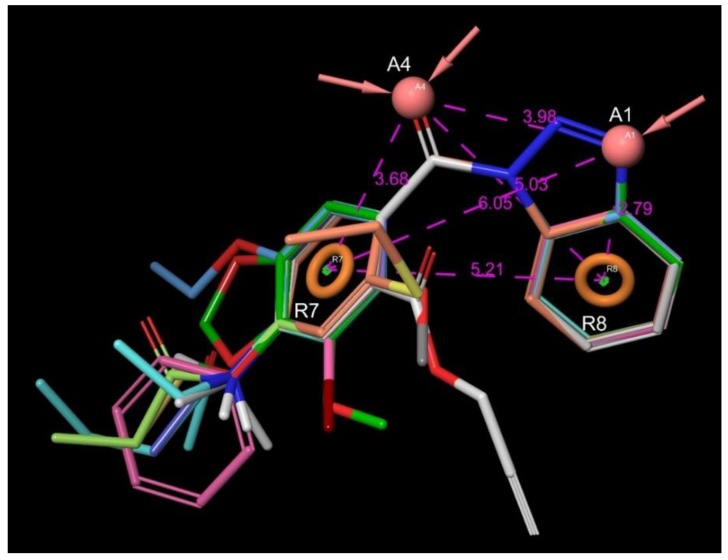
The selected 1,2,3-benzotriazole model (AARR_3). One of the aromatic groups (R8) and the two acceptor groups (A1 and A4) groups are planar while the plane of the second aromatic group (R7) is slightly bent.

**Figure 3:**
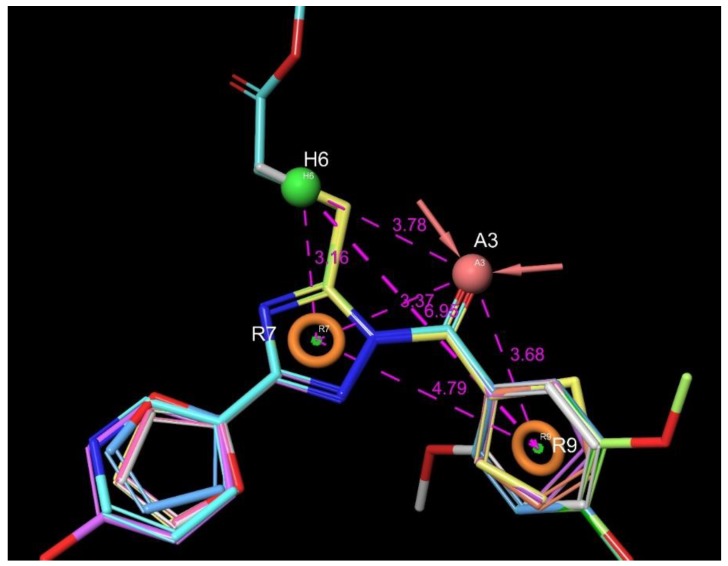
The selected 1,2,4-triazole model (AHRR_1). One of the aromatic groups (R7) and the acceptor group (A3) and the hydrophobic group (H6) are planar while the plane of the second aromatic group (R9) is twisted.

**Figure 4:**
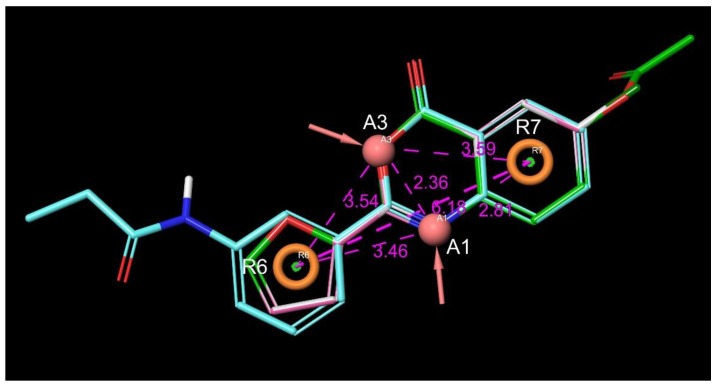
The selected 3,1-benzoxazin-4-one model (AARR_3). Unlike the other two models, this structure is planar, the two acceptor groups (A1 and A3) are located more close to each other while the two aromatic groups (R6 and R7) are located further away from each other.

The Top5 hypotheses of each set was tested during the final validation with the Hypothesis Validation task of Schrödinger’s Phase module using the Enrichment Viewer Panel involving the above mentioned inactive decoy set. Receiver Operator Characteristic (ROC) area under the curve diagrams were generated based on the false positive and true positive rates of the modes and BEDROC160.9 scores (a modified BEDROCK score recommended for hypothesis selection) enrichment factor for recovering 1% of the known actives (EF1%) scores were also calculated together with the ROC scores. Hypothesis with the best scores were selected as final models of the three (1,2,3-benzotriazole, 1,2,4-triazole and 3,1-benzoxazin-4-one) sets and utilized in the screenings (Figure 2, Figure 3 and Figure 4 and Table 9, Table 10 and Table 11).

Common models including all four types of compounds (1,2,3-benzotriazoles, 1,2,4-triazoles, 3,1-benzoxazin-4-ones and imidazoles – 50 compounds altogether) has been also developed (see Supplementary Information). Only one of these model had a full match for all the active compounds (13) while the other two common hypotheses (ARRR_1 and AARR_1) had only 10 out of the 13 active compounds fully matched. The best common model (with all active compounds matching) was not fundamentally different from the AARR_3 (3,1-benzoxazin-4-one) model but it’s survival- and BEDROC scores however were significantly lower (see Supplementary Information) and therefore we have decided not to use them in our virtual screening campaign.

The pharmacophore based screening resulted in six active compounds out of the purchased 21 (hit rate = 28.5%). Such library contained 1,2,3-benzotriazoles (2); imidazoles (2), 3,1-benzoxazin-4-ones (5) as known chemotypes, while 12 compounds belonged to previously not identified chemotypes among the C1s inhibitors. Out of the novel scaffold-bearing compounds two compounds showed inhibition against C1s: One compound had a thieno[2,3-*d*][1,3]oxazin-4-one core (#17; 549 nM) and another belonged to 1,3-benzoxazin-4-ones (#21; 241 nM), which can be considered as a bioisoster of the known “reverse” 3,1-benzoxazin-4-one chemotype. The remaining active compounds were 3,1-benzoxazin-4-ones (2) and benzotriazoles (2).

Since only one compound represented each new chemotypes (they were “singletons”) the outcome of the pharmacophore based in silico screening required an extended hit validation. Applying a comprehensive substructure search on the two novel scaffold structures we selected 19 analogues around the thieno[2,3-*d*][1,3]oxazin-4-one core with diverse substitution pattern (close and remore analogues) and we purchased 11 compounds. Similarly, we selected seven analogues around the 1,3-benzoxazin-4-one core and purchased five analogues. As a control we also purchased two compounds with chromen-4-one structures in which N-3 was replaced with a carbon. Hit validation set resulted in three active analogues for the thieno[2,3-*d*][1,3]oxazin-4-one core (best: #18; 845 nM); and two active residues for the 1,3-benzoxazin-4-one chemotype (best: #22; 3.6 μM), which validated the obtained new chemotypes. The hit rate of the combined hit (defined as an activity ≤ 10 μM) validation set was 13% (excluding the unrelated chromen-4-one structures).

In the three screening rounds we tested altogether 89 compounds. We also analyzed the hit rates according to the chemotypes. For the known chemotypes we found: 1,2,3-benzotriazoles: 44%; 1,2,4-triazoles: 26.6%; 3,1-benzoxazin-4-ones: 30% hit rate. For the novel chemotypes we obtained 1,3-benzoxazin-4-one: 33.3%; thieno[2,3-*d*][1,3]oxazin-4-one: 16.6%, which proves that the hit validation of these novel scaffolds was successful.

### 3.1. Structure–Activity Relationship of the Chemotypes

#### 3.1.1. 1,2,3-Benzotriazoles

**Scheme 5:**
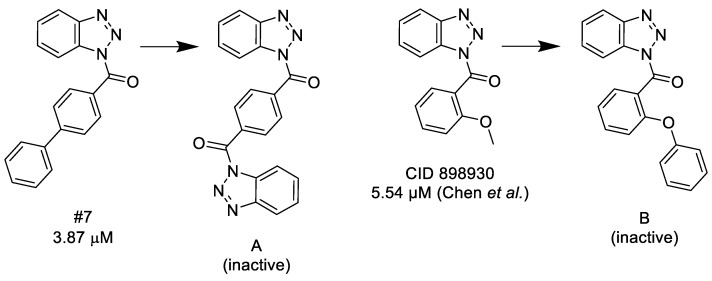
Examples of the 1,2,3-benzotriazoles structure–activity relationship.

The highest number of activity data are available for this chemotype. Recently, Chen et al. [11] attempted a preliminary SAR based on the available PubChem activity data and their in-house activity measurements. We extended the list with eight active and 10 inactive compounds, thus, altogether 20 active compounds and numerous inactive compounds refined the SAR determination (Scheme 5). 

The additional data confirmed mostly the previous findings reported by Chen [11]. The major facts are the following:

(1) A substituted benzoyl group in the *N*-1 position is required for the activity. Unsubstituted benzoyl groups or replacement with an *N*-1 furoyl group is detrimental to the activity.
(2) The position and nature of the functional groups on the benzoyl group have a significant impact on the activity. The *para* position is much favored, followed by the *ortho* position. Substituents in the *meta* position generally diminish or occasionally fully eliminate the activity. However, *meta*/*para* disubstitution somewhat counterbalances this negative effect.
(3) Electron donating groups (amide, ether, thioether) at the *para* position are particularly beneficial. Alkyl groups are weaker in the *para* position, but interestingly bulky alkyl groups (*t*-butyl) look better. Interestingly, while a *meta*/*para* difluoro-substituted compound is relatively good inhibitor, analogous dichloro substitution and *para*-bromo substitution led to inactive compounds. Electron acceptor groups in the *para* position (such as nitro group) eliminate the activity.

From our focused library screening we had some interesting observations regarding the effect of bulk substituents in the *para* and *ortho* positions. The hydrophobic biphenyl group is relatively good, while the benzoyl bridged bis(benzotriazole) compound (1-[4-(1,2,3-benzotriazole-1-carbonyl)benzoyl]-1,2,3-benzotriazole) becomes inactive. Similarly, the *ortho*-methoxy substituted benzoyl is active, while the bulky phenyl ether loses the activity. We also found that changing the benzoyl group to benzyl eliminates the activity. Only one compound (**5**) was found in the ChEMBL biological activity database ((https://www.ebi.ac.uk/chembl) but with unrelated biological activities (FXI, FXII inhibition).

#### 3.1.2. 1,2,4-Triazoles

**Scheme 6:**
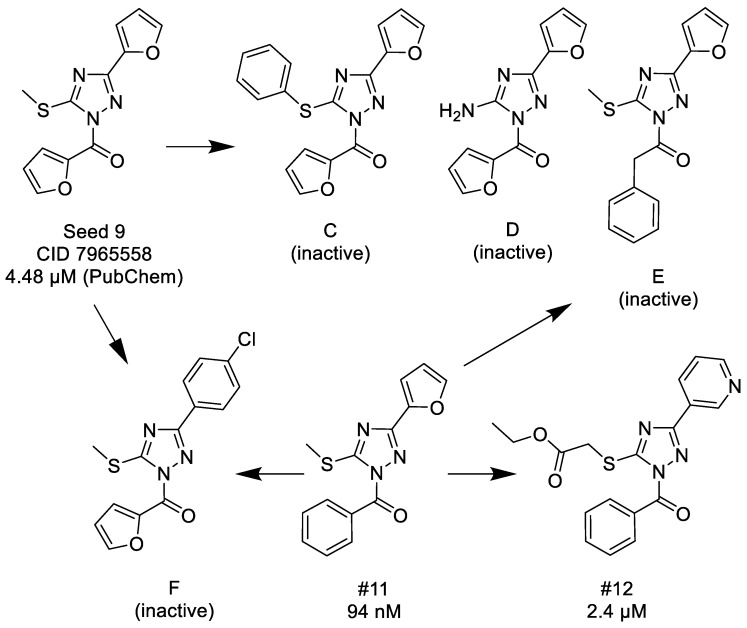
Examples of the 1,2,4-triazoles structure–activity relationship.

There are only few known active C1s inhibitors of this chemotype. The data are available from the PubChem. We tested 15 analogues and found four active compounds, which were among the highest activity compounds. (#9: IC_50_= 12 nM; #10: IC_50_ = 44 nM; #11: IC_50_ = 94 nM). These compounds derived from the 2D similarity search. The measured compounds represented a well-defined chemical space, and thus allowed us to draw some conclusions about the SAR (Scheme 6):

(1) Unsubstituted aroyl (furan-carbonyl, thiophene-carbonyl and benzoyl) groups in the *N*-1 position are beneficial. *Ortho* and *para* substitutions of the *N*-1 benzoyl group (such as methyl, methoxy, and fluoro) have also positive contributions. Replacing the *N*-1 aroyl groups to propionyl, benzyl-carbonyl leads to the loss of the activity.
(2) Replacing the methyl-thio group to primary/secondary amines or bulky aralkyl groups results in inactive compounds.
(3) Changing the C-3 furyl group to thiophenyl (PubChem, CID 4257399; IC_50_ = 880 nM) or 3-pyridyl group (#12; IC_50_ = 2.4 μM) retains the activity, while replacing with *para*-chloro-phenyl leads to inactivation.

Two hit compounds (**9**, **11**) were found in the ChEMBL biological activity database but with various unrelated biological activities (thrombin, FXI, FXII inhibition).

#### 3.1.3. 3,1-Benzoxazin-4-ones

**Scheme 7:**
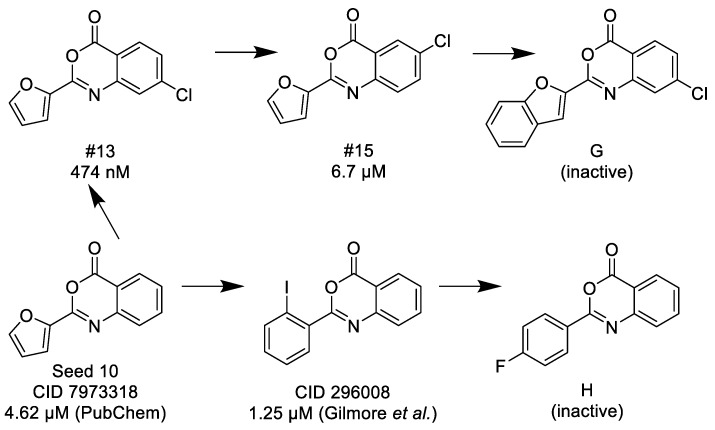
Examples of the 3,1-benzoxazin-4-ones structure – activity relationship.

This chemotype (2-aryl-3,1-benzoxazin-4-ones) was originally reported by Gilmore and co-workers in 1996. The best compound was 2-(2′-iodophenyl)-3,1-benzoxazin-4-one (CID296008, IC_50_= 1.25 μM) [47]. Later the previously cited HTS campaign identified two further hit compounds [11], and one of them was applied as a lead compound (seed #10; see Supplementary Materials) in the 2D similarity search. Ten compounds were tested and four were found active (three compounds had inhibitory activity below 10 μM). The following limited SAR observations were made (Scheme 7):

(1) Halogen (chloro) in the C-7 position is much favored over the C-6 position as well as to the unsubstituted 2-aryl-3,1-benzoxazin-4-ones.
(2) Replacing the furyl group to 2′-iodo-phenyl at the C-2 position improves the activity, while 4′-fluorophenyl group in the same position eliminates the activity.
(3) If the furyl group at the C-2 position replaced with a bulky 2-benzofuryl group the compound loses the activity.

None of the hit compounds was found in the ChEMBL biological activity database.

#### 3.1.4. 1,3-Benzoxazin-4-ones

**Scheme 8:**
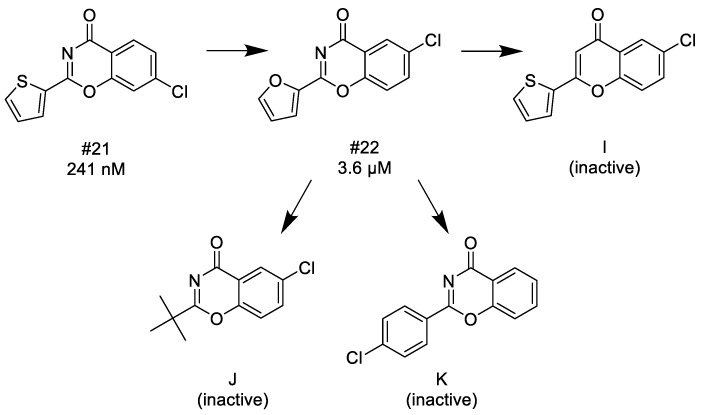
Examples of the 1,3-benzoxazin-4-ones structure–activity relationship.

Altogether six compounds were tested and three were found active (two compounds had inhibitory activities below 10 μM). This novel chemotype is closely related to the previous one, the *N* and *O* changed positions (N: 1 to 3; O: 3 to 1), thus, the two structures can be considered as bioisosteres. This close relationship suggests that the main SAR characteristics should be very similar (Scheme 8). Indeed, we found a very similar behavior to the 3,1-benzoxazin-4-one chemotype. The biological activities of this “reverse” benzoxazine chemotype are comparable but slightly better (ca. two fold):

(1) Since thiophene and furan are interchangeable in many bioactive compounds, therefore, the trend of the decreasing activity between #21 and #22 is comparable to the same direction between #13 and #15, thus, halogen (chloro) in the *C*-7 position is much favored over the *C*-6 position.
(2) Introducing 4′-chlorophenyl group or alkyl groups at the *C*-2 position instead of furyl or thiophenyl groups leads to inactivation.
(3) Finally, if N-3 is replaced with carbon forming the corresponding chromen-4-one, it results in a complete loss of the activity.

None of the hit compounds was found in the ChEMBL biological activity database.

#### 3.1.5. Thieno[2,3-*d*][1,3]oxazin-4-ones

**Scheme 9:**
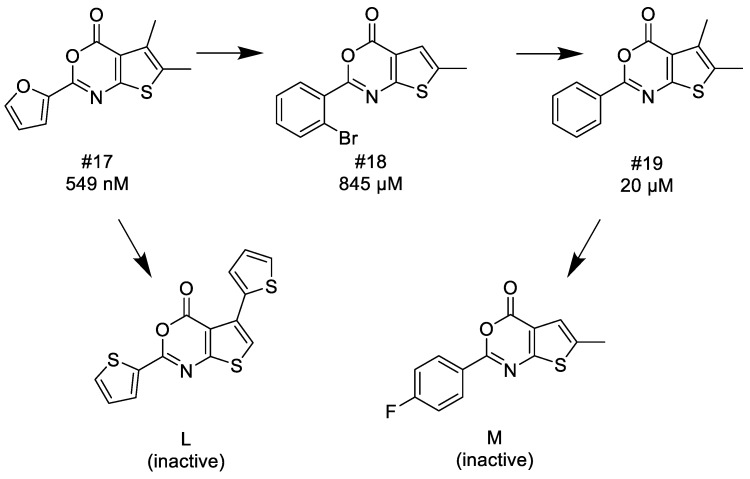
Examples of the thieno[2,3-*d*][1,3]oxazin-4-ones structure–activity relationship.

Altogether 12 compounds were tested and four were found active (two compounds had inhibitory activities below 10 μM). This novel chemotype is also related to the known 3,1-benzoxazin-4-one cores; the fused phenyl group was replaced with thiophene, thus, it is expected that the SAR characteristics should reveal some similarities (Scheme 9):

(1) Similarly to 3,1-benzoxazin-4-ones replacing the furyl group with 2′-halogeno (bromo)-phenyl at the C-2 position retains the activity, while unsubstituted phenyl, 4′-tolyl, 4′-halogeno (fluoro, chloro) substituted phenyl groups in the same position significantly reduce or completely eliminate the activity.
(2) Introducing a bulky (thiophenyl) group into the thiophenyl part of the fused ring leads to complete inactivation.

The hit compound **17** was previously measured for C1s inhibitory activity as reported in the ChEMBL and PubChem (https://pubchem.ncbi.nlm.nih.gov/) but it was claimed to be “inactive” due to inadequate data processing [9]. Another hit compound (**18**) was found in the ChEMBL biological activity database but with unrelated biological activities (FXI, FXII inhibition).

#### 3.1.6. Structural Features of the C1s Selectivity over Factor Xa

Looking at the Factor Xa inhibition of the 20 C1s inhibitor hit compounds we identified the following structural features: Bulk substituents in the *para* position of the phenyl group, which is either isolated (such as in 1,2,3-benzotriazoles and 1,2,4-triazoles) or part of the rings (such as 3,1-benzoxazin-4-one; or thieno[2,3-*d*][1,3]oxazin-4-ones) favors the selectivity over Factor Xa (#4, #6, #7, #13). On the other hand, if substituents are in the *ortho* (or occasionally in *meta*) positions the selectivity is reduced (#3, #9, #22).

## 4. Materials and Methods

### 4.1. Molecular Biology. Protein Expression of C1s

Recombinant C1s and C1r fragments (CCP1-CCP2-SP, the γB catalytic region) containing the serine protease domain were expressed in *E. Coli BL21(DE3)pLysS* host strain (transformed with *pET-17b vector*) in form of inclusion bodies; proteins were isolated, renatured and purified as published earlier [48]. Recombinant C1s, isolated in intact proenzyme form, was activated by limited proteolysis using the recombinant, autoactivated C1r fragment. Activated C1s and C1r were separated using Q Sepharose HP (Amersham Biosciences, Little Chalfont, UK) anion exchange chromatography.

### 4.2. Assay Development

Two end-point assays have been developed for C1s inhibition assays: For screening assays a specific, fluorescent amide substrate Boc-Leu-Gly-Arg-AMC (Sigma, St. Louis, MO, USA), representing the cleavage site sequence of C2 was used with relatively high enzyme concentration (0.2 µM). Assay conditions: 20 mM HEPES (pH 7.4), 200 mM NaCl, 0.05% Tween20, 1% DMSO; 384 microplate format; 10 µL enzyme + 10 µL inhibitor compounds (final concentration: 10 µM); incubation time and temperature: 10 min, RT; then 20 µL substrate solution was added (1 mM final concentration); incubated (45 min, 30 °C) and the fluorescence have been read (355/460 nm; Wallac 1420 Victor2 microplate reader, Perkin Elmer, Waltham, MA, USA). FUT175 (Sigma), a non-specific, highly active serine protease inhibitor has been used for inhibition control.

IC_50_ measurements have been carried out with high concentration of a sensitive thioester substrate (*Z*-Lys-SBzl, Sigma) combined with DTNB thiol reagent (Sigma) and low enzyme concentration (below 1 nM), in the same buffer used for 10 µM screening, in a 96 well microplate format (in 100 µL), using non-binding plates (Greiner, Kremsmünster, Austria). 40 µL enzyme + 10 µL inhibitor compounds were incubated (10 min, RT), and 50 µL substrate solution (1 mM final concentration) was added; incubated for 45 min and finally absorbance was read (405 nm) with a Perkin Elmer Wallac 1420 Victor2 microplate reader. The assay has been validated with FUT175 with IC_50_ = 2 × 10^−9^ M (in contrast with the reported values: 2,9 × 10^−8^ M [4] and 3.2 × 10^−7^ M [49]); revealing high sensitivity of the assay developed for IC_50_ measurements.

### 4.3. Factor Xa (FXa) Assay Development

FXa activity assay has been developed to test specificity of the selected inhibitors. 10 µM one-point screen and IC_50_ measurement have been carried out as follows:

Human isolated Factor Xa protein was purchased from ThermoFisher Scientific (Waltham, MA, USA). An end-point assay was carried out using the fluorescent amide substrate Boc-Leu-Gly-Arg-AMC used for C1s 10 µM screen; assay conditions: 20 mM HEPES (pH 7,8) 100 mM NaCl, 10 mM CaCl_2_ 30 °C, 0.05% TWEEN-20, 1% DMSO; low enzyme concentration (about 1 nM); in a 96 well microplate format (in 100 µL), using non-binding black plates (Greiner). 40 µL enzyme + 10 µL inhibitor compounds were incubated (10 min, RT), and 50 µL substrate solution (500 µM final concentration) was added; incubated for 30 min and finally the fluorescence has been read. Edoxaban (Cayman Chemical, MI, USA), a specific and highly potent FXa inhibitor has been used for inhibition control and for validation of the assay with a measured IC_50_ = 0.649 nM value, according to the published data (Ki of 0.561 nM [50]).

### 4.4. Chemoinformatics Methods

#### 4.4.1. 2D Similarity Selection

One of the key concepts in the ligand-based vs. approaches [51,52] is the Similarity Property Principle, which states that similar molecules should have similar biological properties [53,54]. In other words, a molecule that has not been tested for biological activity but that is structurally similar to an active molecule (reference or seed molecule) is also likely to be active [38]. If determining the similarity between biologically active reference compound and each molecule in a database, followed by ranking the database molecules according to the similarities would lead to potentially active, target-focused libraries.

Similarity search uses 2D fingerprints, i.e., binary strings encoding the presence or absence of a substructure within the molecules [55]. Applying simple 2D fingerprints is often the method of choice [56] particularly when numerous active, reference compounds and multimillion compound databases are available. Its computational efficiency is coupled with demonstrated effectiveness in many comparative studies [57].

Rapid 2D similarity search can be performed on multimillion compound’s databases if structures of active molecules are available therefore, it is a “real time” method compared with the other ligand-based approaches.

Focused library screening often results in a many fold increase in the hit rate compared with random screening of a commercial libraries [14,15]. In many cases virtual screening methods and in vitro HTS were combined [58] and found complementary to each other [40,59,60].

The 2D similarity search can often be refined using physicochemical descriptors in property–based virtual screening.

If most of the parameters of the drug candidate fall into the pre-defined ranges the concerning molecule could be administered orally [61]. The major physico-chemical properties (Mwt, LogP, H-bond donors/acceptors, rotatable bonds and topological polar surface area – tPsa) were determined by the Calculation Suit of InstJChem (ChemAxon Ltd., Budapest, Hungary). The first four parameters are included in the Lipinski’s Rule of 5 [62], while the other two are referred to as the Veber rules [63].

We applied InstJChem software (Version: 16.12.12.0, date: 12.15.2016) for 2D similarity search. InstJChem uses the Chemical Hashed Fingerprints for the 2D similarity search (as discussed earlier). Normally, in the initial similarity search phase a compound was defined as similar, if the Tanimoto coefficient was > 0.65 compared to any reference compound. In the following screening rounds (e.g., in hit validation or hit refinement), we often applied higher Tanimoto similarity cut-off values. Most frequently Tanimoto coefficient [64] is used for measuring similarity [53,65] in spite of its marked size-dependency [66]. It typically yields low similarity values when the reference molecules are relatively small and just lead to few bits set in the fingerprint.

The searchable drug-like chemical space, which is the major target of the similarity search, was composed by existing compounds: non-exclusive commercial libraries were available from the actual edition of the top vendor databases (~5 million compounds): Asinex, ChemBridge, ChemDiv, Enamine, Interbioscreen, Life Chemicals, Specs, UkrOrg, etc. [21,22,23,24,25,26,27,28]. The biologically active chemical space is composed of known, biologically active reference (seed) compounds. Known C1s inhibitors were collected from the available literature, PubChem and various commercial databases.

#### 4.4.2. 3D Modelling

##### X-Ray Structures

In the past two decades, ten X-ray crystallographic structures of human C1s have been published in the literature with various (Table 12) resolutions [29,67,68,69,70,71]. Seven of these structures have not included the catalytic serine protease domain and therefore they were unsuitable for structure-based inhibitor modelling, but the remaining three structures were also problematic mostly because the lack of co-crystalized small molecule inhibitors.

The first human C1s structure has been an *apo* form of the enzyme published by Gaboriaud et al. in 2000 (PDB code 1ELV) with 1.7 Å resolution contained the substrate binding site [29]. This structure seemed to be an obvious choice as it has been used for docking studies by the research group of Subasinghe and Travis [5,6,7].

The second structure that could be considered has been reported by Perry et al. in 2013 (PDB code 4J1Y) as the zymogen form of the enzyme co crystalized with its endogenous substrate, complement C4 with a poor 2.66 Å resolution [69].

The third structure has been recently introduced by Pang et al. in 2017 (PDB code 5UBM), in which the enzyme in complex with the protein inhibitor gigastasin (isolated from from the Giant Amazon Leech, *Haementeria ghilianii*) with a medium resolution of 2.5 Å [70].

##### Ligand Preparation

Small molecule structures were downloaded from ChEMBL open bioactivity database (https://www.ebi.ac.uk/chembl) in SMILES format [72], and converted into 2D structures via the JChem for Excel application (JChem for Office (Excel) 2019.6.0.447, ChemAxon (http://www.chemaxon.com). Ligand preparation was completed with Schrödinger’s LigPrepmodule (Schrödinger Release 2018-2: LigPrep, Schrödinger, LLC, New York, NY, USA, 2018), structures were optimized with OPLS3e force field (Schrödinger LLC, Release 2018-2, OPLS3e, 2018) [73], Epik module was used to the generation of various stereochemistry (if undefined) as well as protonation- and tautomeric forms at pH = 7.4 (Schrödinger LLC, Release 2018-2: Epik, 2018) [74].

##### Protein Preparation and Docking

Protein structures were downloaded from the RCSB Protein Data Bank (https://RCSB.org) [75], readied for docking by Schrödinger’s P Protein Preparation Wizard, standard settings were used (Schrödinger Release 2018-2: Protein Preparation Wizard, 2018) [76]. Docking studies were completed with Schrödinger’s Glide docking software (Schrödinger Release 2018-2: Glide, 2018) [77,78,79]. The doxing box was centered on the catalytic serine (Ser617) and the size of the box was set to 30 × 30 × 30 Å.

Docking studies were completed on both standard precision (SP) extra precision (XP) level with standard settings, no constraints were used. In order to improve the improve the interactions between the binding site residues and the ligands, Induced Fit Docking (IFD) technics were applied on the 1ELV in complex with published active C1s serine protease inhibitors (Schrödinger Release 2018-2: Glide and Prime modules, Schrödinger LLC, 2018) [80]. An H-bond constrain has been applied between the ligand’s amidine groups and the aspartic acid (Asp611) of the binding site thought to be essential in substrate- and inhibitor binding. The optimized IFD complex – after the removal of the constrained ligand – has been used for further docking studies.

#### 4.4.3. Pharmacophore Modeling

The ‘create pharmacophore model with multiple ligands’ option of Schrödinger’s Phase software was used for model building (Release 2018-2: Phase, Schrödinger LLC2018) [81]. After a conformer library generation, a low energy conformer of an active ligand of the set was chosen as reference and its variants of pharmacophore group were aligned with those of the other compounds’ pharmacophore features. Hypotheses were generated using the find best alignment and common features pharmacophore method based on 4 to 6 features with 0.50 difference criterion cutoff and a maximum of 2.0 Å distance tolerance for each features, at least 75% of active ligands should have match. These model hypotheses than were scored and ranked by PhaseHypoScore, an optimized weighted sum of Survival and (Boltzmann-enhanced Discrimination Receiver Operator Characteristic area under the curve (BEDROC) score [82].

## 5. Conclusions

The CS is associated with various diseases such inflammation or auto-immune diseases. Complement system-targeted drugs could provide novel therapeutic interventions against the above diseases. C1s is an attractive target since it blocks the system at an early stage of the complement cascade. Designing C1s inhibitors is particularly challenging since the inhibitors form a very distinct, narrow bioactive chemical space as well as achieving selectivity over the other serine proteases. The typical architecture of a small molecule inhibitor of C1s contains a heterocyclic amidine (or guanidine), however, discovery of non-amidine inhibitors might have high value particularly if identifying novel chemotypes and/or achieving improved selectivity.

For biological screening the C1s protein was expressed, two inhibition assays (fluorescent and photometric) were developed and validated. The focused libraries were screened first at 10 µM concentration and the IC_50_ values were determined for the most active compounds.

We attempted to apply various virtual screening approaches to identify C1s focused libraries that lack the amidine/guanidine functionalities, then the libraries were evaluated by in vitro biological assays. While 3D structure-based methods were not suitable for virtual screening, and 2D similarity search did not lead to novel chemotypes, pharmacophore model generation allowed us to identify two novel chemotypes with submicromolar activities. In three screening rounds we tested altogether 89 compounds and identified 20 hit compounds (<10 μM activities; overall hit rate: 22.5%). The highest activity compound was 12 nM (1,2,4-triazole), while for the newly identified chemotypes 241 nM and 549 nM, respectively. For selectivity measurements the hit compounds were screened for Factor Xa inhibition, and six compounds showed excellent selectivity. Further investigation of the compounds in the complement system is under progress.

## Figures and Tables

**Table 1:** Hit compounds derived from the 1,2,3-benzotriazole chemotype. We considered hits if the inhibitory activity was <10 μM. In some cases we display compounds with weak activities (≤50 μM; *in italics*).

Entry	Structure	IDNUMBER	IC_50_ C1s; µM	LogP	TPSA	H Bond Acceptors	H Bond Donors	Rotatable Bonds	Lipinski Rule of 5 (4 of 4)	Chemoinformatics
1	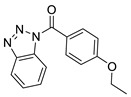	BAS 00784557	0.083	2.6744	57.01	4	0	3	true	2D similarity
2	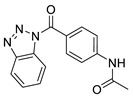	2616-0473	0.091	1.713	76.88	4	1	2	true	2D similarity
3	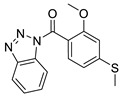	3502-2773	0.24	2.9458	57.01	4	0	3	true	Pharmacophore
4	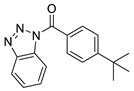	8008-1157	1.30	4.0203	47.78	3	0	2	true	2D similarity
5	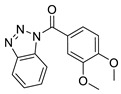	4130-3864 (CHEMBL-143333)7	1.85	2.1599	66.24	5	0	3	true	2D similarity
6	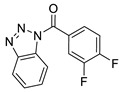	8012-0883	2.16	2.7607	47.78	3	0	1	true	Pharmacophore
7	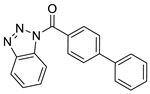	BAS 00785517	3.867	4.1225	47.78	3	0	2	true	2D similarity
8	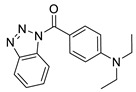	3455-3034	5.90	3.2969	51.02	4	0	4	true	2D similarity

**Table 2:** Hit compounds derived from the 1,2,4-triazole chemotype.

Entry	Structure	IDNUMBER	IC_50_ C1s; µM	LogP	TPSA	H Bond Acceptors	H Bond Donors	Rotatable Bonds	Lipinski rule of 5 (4 of 4)	Chemoinformatics
9	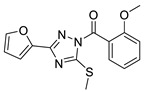	BAS 07161957 (CHEMBL-490106)	0.012	3.45	70.15	4	0	4	true	2D similarity
10	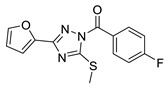	BAS 07161950	0.044	3.79	60.92	3	0	3	true	2D similarity
11	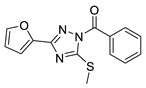	BAS 07161949 (CHEMBL-490107)	0.094	3.65	60.92	3	0	3	true	2D similarity
12	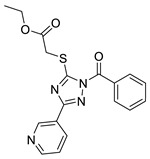	ASN 01365717	2.4	2.97	86.97	5	0	7	true	2D similarity

**Table 3:** Hit compounds derived from the 3,1-benzoxazin-4-one chemotype.

Entry	Structure	IDNUMBER	IC_50_ C1s; µM	LogP	TPSA	H Bond Acceptors	H Bond Donors	Rotatable Bonds	Lipinski Rule of 5 (4 of 4)	Chemoinformatics
13	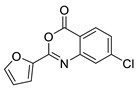	3226-0357	0.474	3.02	51.80	2	0	1	true	Pharmacophore
14	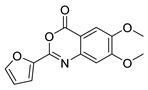	Z55930777	6.44	2.10	70.26	4	0	3	true	Pharmacophore
15	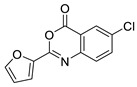	BAS 01170083	6.7	3.02	51.80	2	0	1	true	2D similarity
16	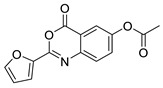	*4334-1472* *(CHEMBL-1449374*	*29*	*2.02*	*78.10*	*3*	*0*	*3*	*true*	*2D similarity*

**Table 4:** Hit compounds derived from the thieno[2,3-*d*][1,3]oxazin-4-one chemotype.

Entry	Structure	IDNUMBER	IC_50_ C1s; µM	LogP	TPSA	H Bond Acceptors	H Bond Donors	Rotatable Bonds	Lipinski Rule of 5 (4 of 4)	Chemoinformatics
17	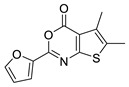	STOCK1S-76323 (CHEMBL-1333976)	0.549	3.52	51.80	2	0	1	true	Pharmacophore
18	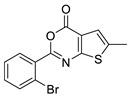	STOCK3S-12710 (CHEMBL-1501165)	0.845	4.72	38.66	2	0	1	true	Hit validation
19	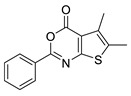	*STOCK2S-97295* *(CHEMBL-1441606)*	*~20*	*4.46*	*38.66*	*2*	*0*	*1*	*true*	*Hit validation*
20	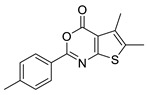	*STOCK2S-12571*	*~50*	*4.98*	*38.66*	*2*	*0*	*1*	*true*	*Hit validation*

**Table 5:** Hit compounds derived from the 1,3-benzoxazin-4-one chemotype.

Entry	Structure	IDNUMBER	IC_50_ C1s; µM	LogP	TPSA	H Bond Acceptors	H Bond Donors	Rotatable Bonds	Lipinski Rule of 5 (4 of 4)	Chemoinformatics
21	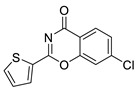	Z55992821	0.241	3.20	38.66	2	0	1	true	Pharmacophore
22	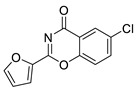	Z55992803	3.6	2.35	51.80	2	0	1	true	Hit validation
23	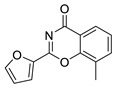	*Z55992807*	*41*	*2.26*	*51.80*	*2*	*0*	*1*	*true*	*Hit validation*

**Table 6:** Factor Xa inhibition of the 20 C1s inhibitor hit compounds. (Compounds with remaining activities >80 % are shown in bold).

Entry	Factor Xa 10 µM Remaining Activity %	Entry	Factor Xa 10 µM Remaining Activity %
1	30.3	11	26.02
2	43.1	**12**	**85.4**
3	4.93	**13**	**98.6**
**4**	**86.2**	14	61.4
5	48.3	15	82
**6**	**92.3**	16	77.6
**7**	**92.1**	**17**	**100**
8	76.5	18	46.1
9	2.39	21	53.6
10	78.1	22	0.14

**Table 7:** Biological data, docking scores and emodel scores of the non-amidine C1s inhibitors obtained by the 2D similarity search.

Entry	IC_50_ (µM)	Glide Docking Score	Glide Emodel Score
1	0.083	−6.404	−58.321
2	0.091	−6.866	−65.171
4	1.300	−7.003	−60.058
5	1.850	−6.791	−59.201
7	3.870	−7.558	−65.852
9	0.012	−6.592	−62.262
10	0.044	−6.099	−54.519
11	0.094	−6.993	−60.231

**Table 8:** Biological data, docking scores and emodel scores of selected non-amidine literature inhibitors (Chen et al., [11]).

Compound ID	IC_50_ (µM)	Glide Docking Score	Glide Emodel Scores
17178137	11.0	−5.538	−53.585
4951143	19.1	−5.595	−57.808
2986934	0.34	−6.892	−67.866
710644	1.09	−6.556	−55.670
5146207	>50	−6.495	−54.781
807111	>50	−5.909	−58.543
1107361	>50	−6.621	−67.010
827004	3.04	−7.022	−55.173
4957387	32.9	−6.737	−60.524
898930	5.54	−6.733	−57.680

**Table 9:** Top 5 pharmacophore hypotheses for the 1,2,3-benzotriazole set. A–acceptor group; H–hydrophobic group; R–aromatic group.

Pharmacophore Model	PhaseHypo-Score	Survival Score	Selectivity Score	BEDROC160.9 (from Validation)	ROC	EF1%
ARRR_1	1.319	5.317	1.219	0.67	0.63	2.78
ARRR_2	1.318	5.305	1.211	0.74	0.51	2.78
AARR_1	1.315	5.247	1.150	0.67	0.79	2.78
AARR_2	1.314	5.236	1.139	0.67	0.80	2.78
AARR_3 *	1.314	5.231	1.141	0.92	0.81	5.56

* Hypothesis selected as best 1,2,3-benzotriazole model.

**Table 10:** Top 5 pharmacophore hypotheses for the 1,2,4-triazole set. A–acceptor group; H–hydrophobic group; R–aromatic group.

Pharmacophore Model	PhaseHypoScore	Survival Score	Selectivity Score	BEDROC160.9 (from Validation)	ROC Score	EF1%
AHRR_1 *	1.283	5.175	1.358	0.52	0.40	2.78
AAAHRR_1	1.259	5.833	2.034	0.29	0.09	2.78
AAAHRR_2	1.258	5.828	2.026	0.14	0.11	0.00
AAAHRR_3	1.258	5.826	2.029	0.11	0.11	0.00
AHRRR_1	1.249	5.665	1.87	0.04	0.23	0.00

* hypothesis selected as best 1,2,4-triazole model.

**Table 11:** Top 5 pharmacophore hypotheses for the 3,1-benzoxazin-4-one set. A–acceptor group; R–aromatic group.

Pharmacophore Model	PhaseHypoScore	Survival Score	Selectivity Score	BEDROC160.9 (from validation)	ROC	EF1%
AAARR_1	1.288	4.808	1.373	0.86	0.62	5.56
ARRR_1	1.279	4.646	1.214	0.86	0.52	5.56
AARR_1	1.274	4.574	1.142	0.86	0.76	5.56
AARR_2	1.274	4.562	1.131	0.86	0.66	5.56
AARR_3 *	1.273	4.550	1.118	0.86	0.84	5.56

* hypothesis selected as best 3,1-benzoxazin-4-one model.

**Table 12:** Published protein C1s X-ray crystallography structures and their domain coverage.

Domains	Position	Length (aa)	X-Ray Structures									
			1ELV *	1NZI	4J1Y *	4LMF	4LOR	4LOS	4LOT	6F1C	6F1H	5UBM *
signal peptide	1–15	15	x	x	x	x	x	x	x	x	x	x
CUB 1	16–130	115	−	+	−	+	+	−	−	+	+	−
EGF-like	131–172	42	−	+	−	+	+	−	−	+	+	−
CUB 2	175–290	116	−	−	−	+	+	+	+	+	+	−
Sushi/CCP1	292–356	65	−	−	+	−	-	+	+	−	−	+
Sushi/CCP2	357–423	67	+	−	+	−	−	-	+	−	−	+
SP (peptidase)	438–688	243	+	−	+	−	−	−	−	−	−	+

* Structures may be suitable for modelling.

## Supplementary Materials

The following are available online, Figure S1. Selected concentration dependency curves (for C1s, Factor Xa); Figure S2. The common pharmacophore models; Table S1a. Seed compounds for 2D similarity search (amidines and guanidines); Table S1b. Seed compounds for 2D similarity search (non-amidines and non-guanidines); Table S2. Biological results of all purchased compounds (2D selection) (only the active compounds are numbered); Table S3. Biological results of all purchased compounds (pharmacophore selection); Table S4. Biological results of all purchased compounds (hit validation); Table S5. Biological data and docking scores of the non-amidine literature inhibitors; Table S6. Pharmacophore based virtual screening of commercial databases; Table S7. Biological data and docking scores of the thiopheneamidine inhibitors; Table S8. Parameters of the common pharmacophore models; Scheme S1. Selected bioisosteric replacements for amidines and guanidines, generated by Bioster 4.0.
