# Supplementary material for: Design and Selection of Novel C1s Inhibitors by In Silico and In Vitro Approaches
Source: Molecules. 2019 Oct 9;24(20):3641. doi: 10.3390/molecules24203641 (PMC6832932; doi:10.3390/molecules24203641)
Supplement: Supplementary file 1 [file molecules-24-03641-s001.pdf]

SUPPLEMENTARY MATERIAL

Table S1a. Seed compounds for 2D similarity search (part 1.)

CdId	Structure	Mol Weight	Formula	PUBCHEM_SID	LogP	IC50 (micro M)
1	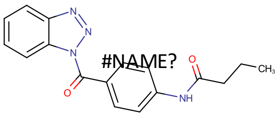 #NAME?	308.341	C17H16N4O2	4 258 988	2.86	0.38
2	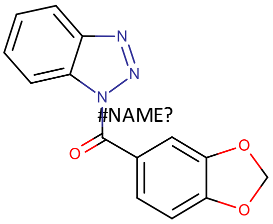 #NAME?	267.244	C14H9N3O3	7 977 382	2.1	0.85
3	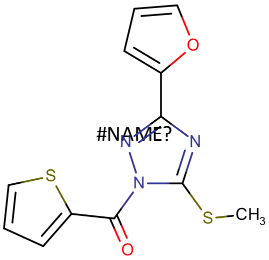 #NAME?	291.34	C12H9N3O2S2	4,257,399	3.56	0.88
4	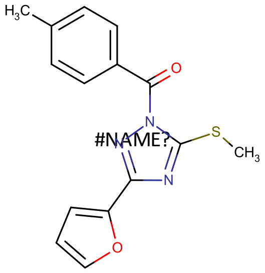 #NAME?	299.35	C15H13N3O2S	7 965 333	4.15	0.9
5	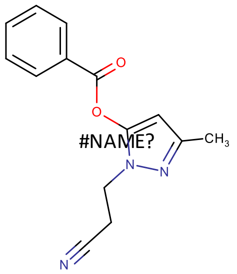 #NAME?	255.277	C14H13N3O2	3 717 586	2.06	2.14

Table S1b. Pubchem seed compounds for 2D similarity search (part 2.)

CdId	Structure	Mol Weight	Formula	PUBCHEM_SID	LogP	IC50 (micro M)
6	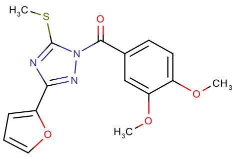	345.37	C16H15N3O4S	4 255 157	3.25	2.37
7	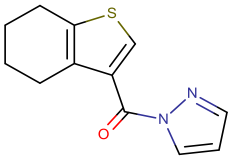	232.3	C12H12N2OS	4 260 000	2.82	3.83
8	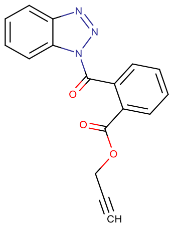	305.293	C17H11N3O3	857 178	2.71	4.26
9	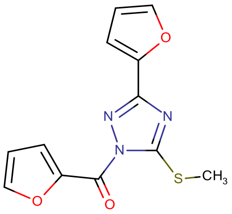	275.28	C12H9N3O3S	7 965 558	2.7	4.48
10	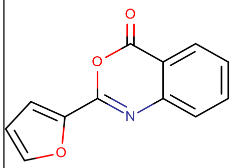	213.192	C12H7NO3	7 973 318	2.42	4.62
11	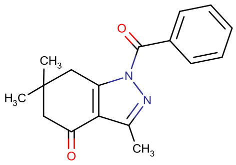	282.343	C17H18N2O2	4 263 151	2.24	5.35
12	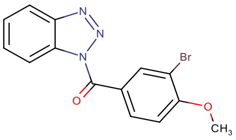	332.157	C14H10BrN3O 2	4 263 449	3.09	5.59

Table S2. Biological results of all purchased compounds (2D selection)

to be added

Table S3. Biological results of all purchased compounds (pharmacophore selection)

to be added

Table S4. Biological results of all purchased compounds (hit validation)

to be added

Figure S1. Selected concentration dependency curves (for C1s, Factor Xa)

Samples: C1s: FUT-175 (reference); #1, #9, #11, #2, #10, #15, #13, #3, #21, #18, #22, #17, #14, Factor Xa: Edoxaban, #9, #3, #22.

C1s assay; IC50 measurements

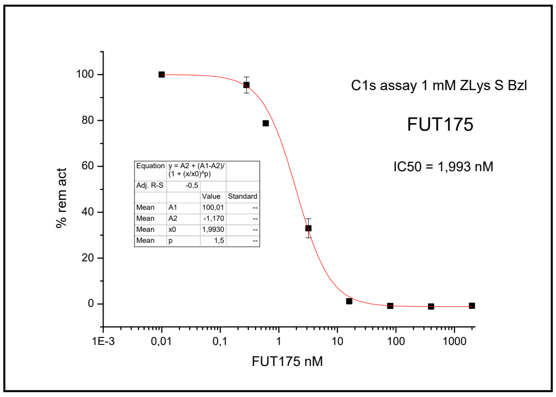

FUT175 ZLyzSBzl 190712

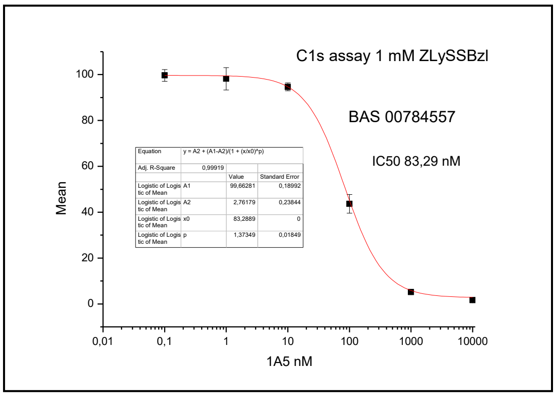

1A5 ZLyzSBzl 180817

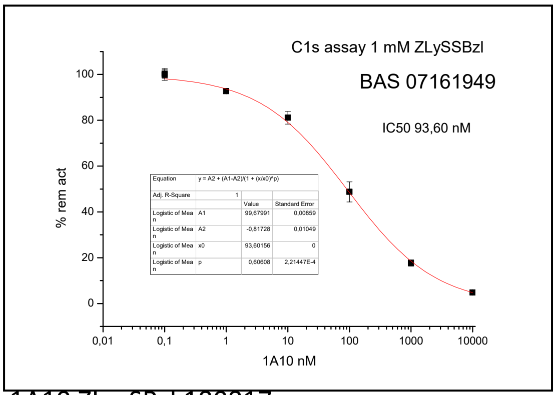

1A10 ZLyzSBzl 180817

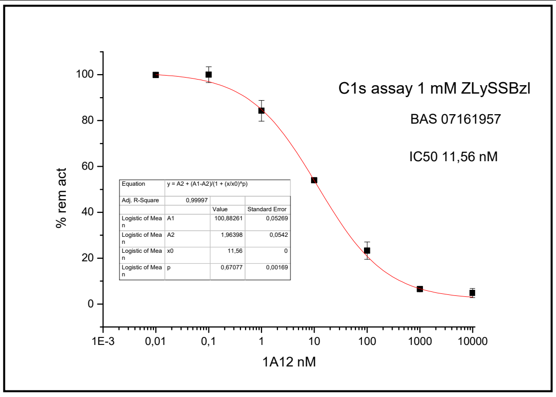

1A12 ZLyzSBzl 180817

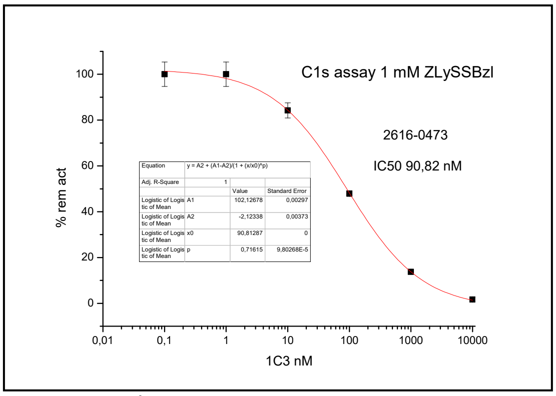

1C3 ZLyzSBzl 180817

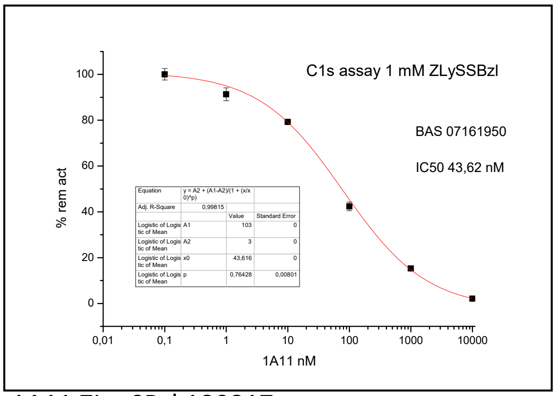

1A11 ZLyzSBzl 180817

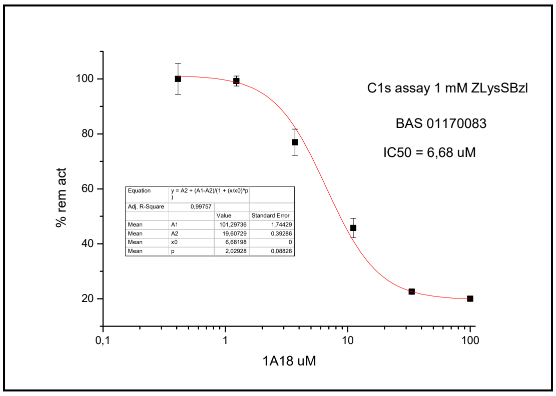

1A18 ZLysSBzl 180829

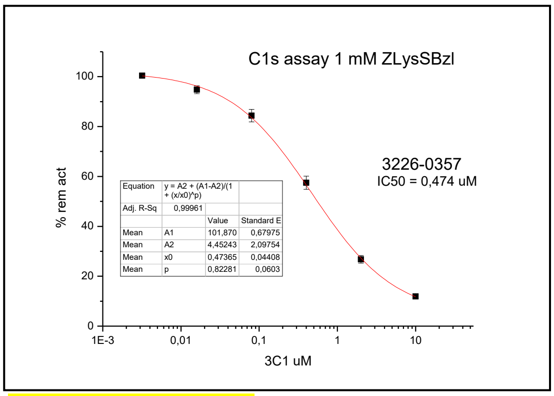

3C1 ZLysSBzl 190807

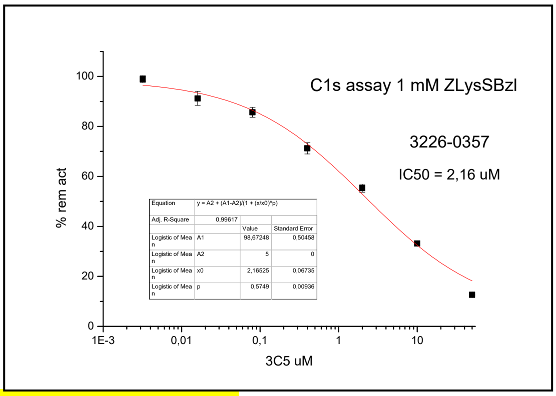

3C5 Z LysSBzl 190807

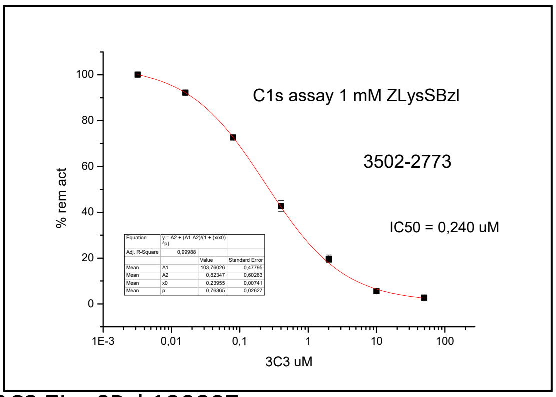

3C3 ZLyzSBzl 190807

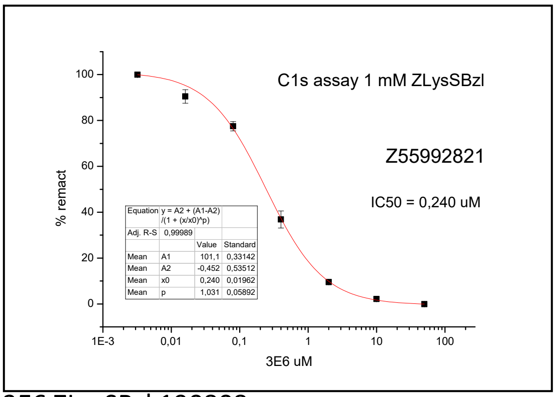

3E6 ZLyzSBzl 190808

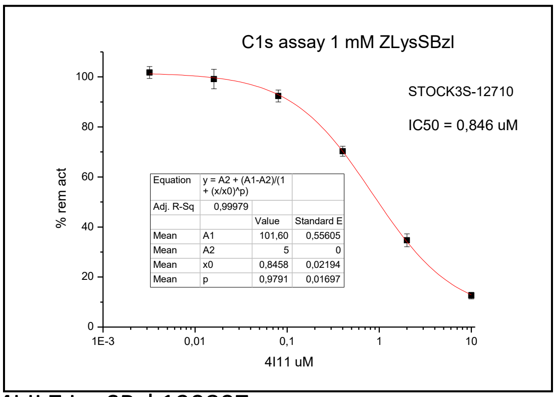

4I II Z LysSBzl 190807

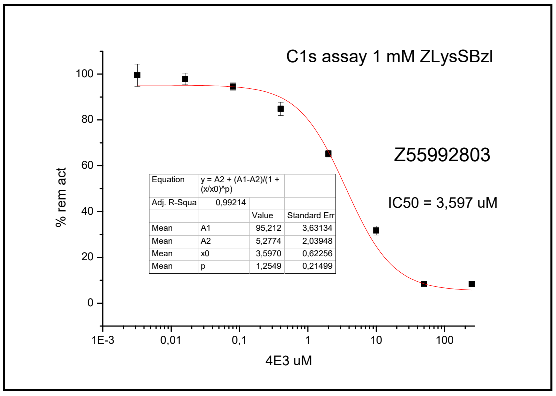

4E3 Z LysSBzl 190807

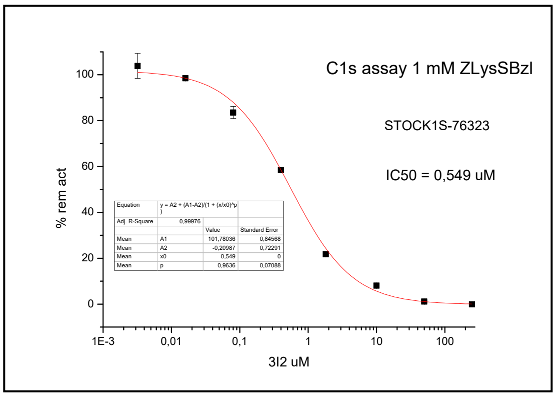

3I2 Z LysSBzl 190807

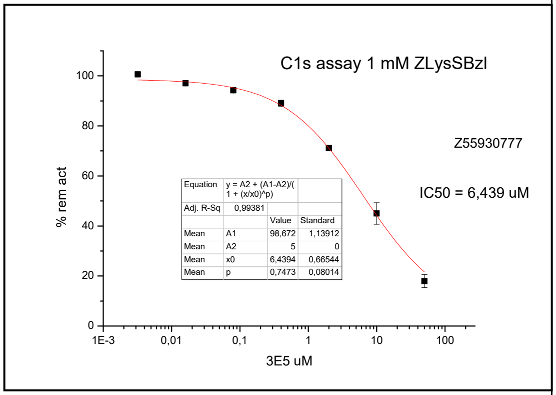

3E5 Z LysSBzl 190807

Fxa assay; IC50 measurements

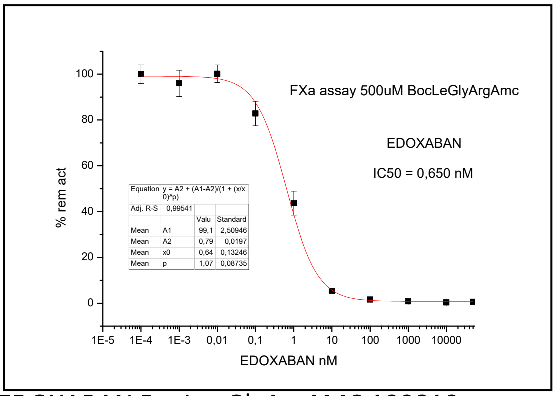

EDOXABAN BocLeuGlyArgAMC 190810

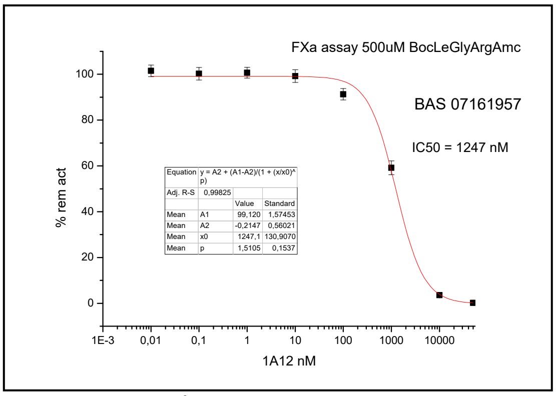

1A12 BocLeuGlyArgAMC 190810

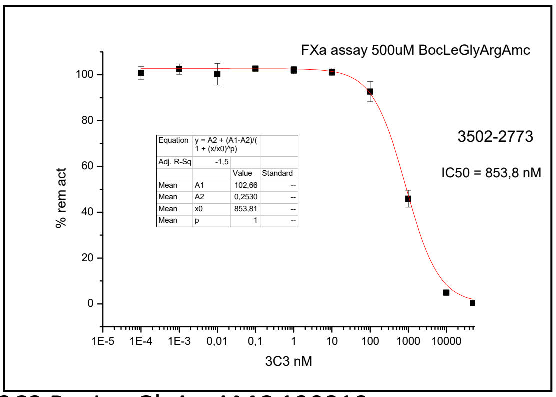

3C3 BocLeuGlyArgAMC 190810

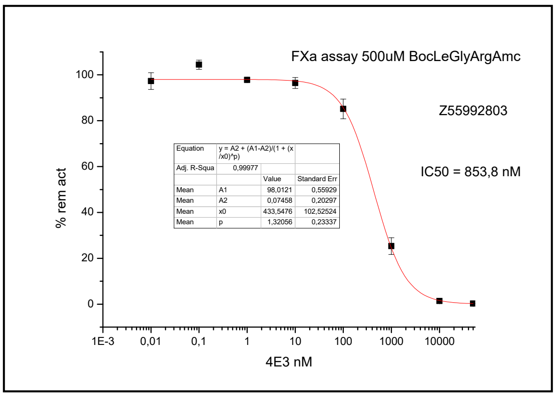

4E3 BocLeuGlyArgAMC 190810

Table S5. Biological data and docking scores of the non-amidine literature inhibitors (Chen *et al*, 2018 [1]) (full list)

**Compound ID**	**IC_{50} (μM)**	**Glide docking score**
17178137	11.0	-5.538
4951143	19.1	-5.595
2986934	0.34	-6.892
710644	1.09	-6.556
5146207	>50	-6.495
807111	>50	-5.909
1107361	>50	-6.621
827004	3.04	-7.022
4957387	32.9	-6.737
898930	5.54	-6.733
17178134	23.1	-5.801
17178138	42.6	-5.484
17131127	>50	-5.420
834536	>50	-6.729
693001	>50	-6.494
792914	>50	-6.012
570059	>50	-6.944

Table S6. Pharmacophore based virtual screening of commercial databases

Total number of structures in the Phase DB	**Number of hits with pharmacophore HTS**		
	**1,2,3-Benzotriazole AARR_3 model**	**1,2,4-Triazole AHRR_1 model**	**3,1-Benzoxazin-4-one AARR_3 model**
679420 (from 445457)	49164	65482	21847

Table S7. Biological data and docking scores of the thiopheneamidine inhibitors (Subasinghe *et al*, 2004 [2])

Compound ID	# in article	K_i (μM)	Glide docking score
CHEMBL321519	3	>20	-8.575
CHEMBL105102	4	>20	-8.965
CHEMBL104368	5	>20	-8.323
CHEMBL441273	6	3	-9.999
CHEMBL106071	7	6	-9.633
CHEMBL105652	8	>28	-7.914
CHEMBL103840	9	12.5	-10.194
CHEMBL322207	10	14.4	-9.021
CHEMBL28875	11	0.45	-9.242
CHEMBL29290	12	0.49	-9.921
CHEMBL31549	13	0.7	-9.858
CHEMBL285818	14	1.02	-9.805
CHEMBL28723	15	0.87	-9.896
CHEMBL287292	16	0.52	-9.987
CHEMBL283859	17	4.7	-9.931
CHEMBL29269	18	1.06	-8.422
CHEMBL281899	19	0.56	-10.076
CHEMBL322672	20	3.25	-9.750
CHEMBL102417	21	0.46	-10.066
CHEMBL29304	22	0.42	-9.884
CHEMBL286243	23	0.436	-10.201
CHEMBL418023	24	15.400	-10.289
CHEMBL29006	25	1.0	-10.432
CHEMBL28618	26	0.90	-10.067
CHEMBL283080	27	0.85	-10.176
CHEMBL28949	28	0.85	-9.970
CHEMBL29205	29	0.47	-9.762
CHEMBL286498	30	0.96	-8.081
CHEMBL286267	31	0.64	-9.947
CHEMBL285142	32	3.0	-10.035
CHEMBL413764	33	0.86	-9.231
CHEMBL318576	46	0.33	-9.180
CHEMBL106388	47	0.19	-9.636

CHEMBL322834	48	0.15	-10.353
**CHEMBL319394**	**49**	**0.06**	**-10.019**
CHEMBL321341	50	0.09	-9.891
CHEMBL101698	51	1.50	-10.252
CHEMBL102835	52	0.53	-7.223
CHEMBL317917	53	0.07	-9.908

References:

1. Chen, J. J.; Schmucker, L. N.; Visco, D. P., Pharmaceutical Machine Learning: Virtual High-Throughput Screens Identifying Promising and Economical Small Molecule Inhibitors of Complement Factor C1s. *Biomolecules* **2018,** 8, (2).
2. Subasinghe, N. L.; Ali, A.; Illig, C. R.; Rudolph, M. J.; Klein, S.; Khalil, E.; Soll, R. M.; Bone, R. F.; Spurlino, J. C.; DesJarlais, R. L.; Crysler, C. S.; Cummings, M. D.; Morris, P. E.; Kilpatrick, J. M.; Babu, Y. S., A novel series of potent and selective small molecule inhibitors of the complement component C1s. *Bioorg Med Chem Lett* **2004,** 14, (12), 3043-3047.
